# Efficient conversion of furfural to cyclopentanol over lignin activated carbon supported Ni–Co catalyst

**DOI:** 10.1039/d2ra00016d

**Published:** 2022-04-19

**Authors:** Qi Guo, Xinglong Hou, Wei Xu, Junli Liu

**Affiliations:** Institute of Chemical Industry of Forest Products, Chinese Academy of Forestry, Key Lab. of Biomass Energy and Material, National Engineering Lab. for Biomass Chemical Utilization, Key and Open Lab. on Forest Chemical Engineering SFA Nanjing 210042 Jiangsu Province China liujunli1974@126.com; Co-Innovation Center of Efficient Processing and Utilization of Forest Resources, Nanjing Forestry University Nanjing 210037 China

## Abstract

Ni_3_Co_1_/ELAC catalyst, prepared by the enzymatically hydrolyzed lignin activated carbon as a carrier and a 3 : 1 ratio content of nickel and cobalt, can selectively convert furfural to cyclopentanol (CPL) in aqueous solution. We used activated carbon prepared by the phosphoric acid method as the carrier, and investigated the effect of the carrier on the catalyst activity. The ratio of bimetal (Ni, Co) content and reaction conditions (reaction temperature, reaction time, initial H_2_ pressure) have also been investigated in the furfural hydrogenation. With the optimal Ni_3_Co_1_/ELAC catalyst, the conversion rate of furfural and the selectivity of CPL were 100% and 94.1%, respectively. In this process, some important catalysts were studied by XRD, XPS, ICP-AES, BET and TEM characterization. Through experimental results and other people's research, we deduced a reasonable reaction path and verified it by replacing the reaction substrate and solvents. Finally, the experiment proved that the formation of CPL by furfural required the occurrence of a rearrangement reaction and the participation of aqueous solution.

## Abstract 2

Ni_3_Co_1_/ELAC catalyst, prepared from enzymatically hydrolyzed lignin activated carbon as a carrier and a 3 : 1 ratio content of nickel and cobalt, can selectively convert furfural to cyclopentanol (CPL) in aqueous solution.

## Graphical abstract

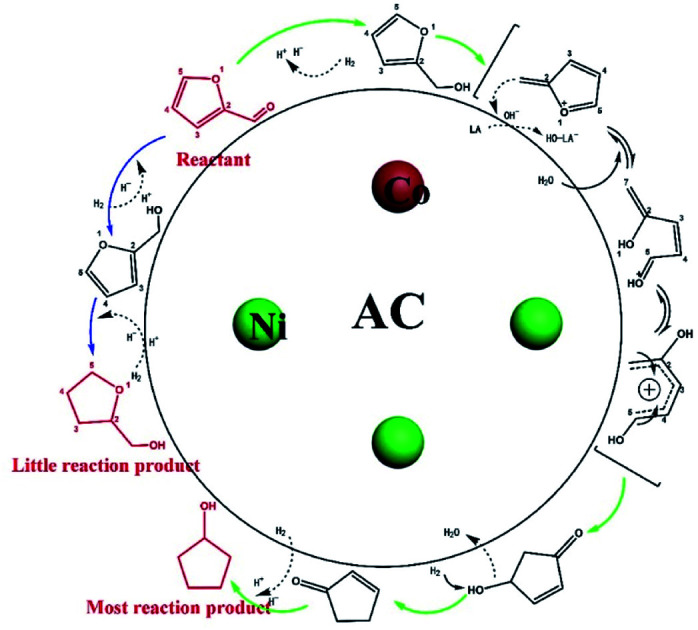

## 1 Introduction

The increase in environmental protection awareness and the massive consumption of fossil fuels have greatly affected renewable biomass resources, which have attracted great attention of many people as an alternative to fossil fuels. Lignocellulose biomass consisting of cellulose (40–50%), hemicellulose (25–35%) and lignin (15–20%) is the most abundant renewable biomass.^1^ One of the challenges of lignocellulose biomass conversion is to obtain target oxygen-containing chemicals, which are more useful sources than fossil fuel because they have higher added value and can be converted into compounds with different functional groups in their production.^2^ Furfural is an important natural precursor to furan-based chemicals, obtained by the catalyzed dehydration of sugars from xylose, extracted from hemicellulose, and has the potential to become a major renewable platform chemical for the production of biochemicals and biofuels.^3,4^ Furfural, as an oxygen-containing functional group compound, is inappropriate as a transportation fuel. So, the improvement of partial or total elimination of the oxygenated functionalities is requisite. Furfural can be converted, through catalytic hydrogenation, into a variety of products of concern, such as furfuryl alcohol (FOL), tetrahydrofurfuryl alcohol (THFOL), cyclopentanone (CPO)^5^ or cyclopentanol^6^ (CPL), which is the main hydrogenated products and widely applied for preparation of fungicides, fragrance chemicals.^7^

However, selective hydrogenation of furfural is a great challenge because there are many different functionalities such as furan ring and C=O bands, which can participate in the reaction resulting in low yield to the desired product and increase the cost for its purification.^8^ So appropriate catalysis plays a central role in the reaction. The high selectivity of the catalyst depends on its supported metal and its carrier, and the ratio.^9^ In the past few years, various metals,^10^ both noble metals (Au, Ir, Pt, Pd, Ru and Rh) and non-noble metals (Ni, Co, Cu, Mo and Fe) have been studied and applied in the hydrogenation reaction of furfural.^11–13^ Though non-noble metals are of lower catalytic activity than noble metals, the research on non-noble catalysts is more valuable and necessary in conversion of FOL. For example, Zhou's group^14^ designed an efficient 30 wt% nickel-based catalyst with HNO_3_-pretreated carbon nanotube (*x*% Ni/CNT, *x* represents the Ni loading amount). They showed the conversion of furfural was up to 96.5% over 30 wt% Ni/CNT and obtained a yield of 83.6%. However, the experiment needed to be carried out under 5 MPa to achieve the above result. Later the same group designed a bimetallic catalyst to improve the experiment.^15^ CPL could also be obtained with a conversion of more than 99% and a yield of 96% with Ni_1_Co_1_/C under 2.0 MPa H_2_ pressure. Experiments have proved that under the same conditions, bimetal has higher conversion rate and selectivity than single metal. Another important part of the catalyst is the carrier. The carrier can not only act as a supporter but also prevent a large amount of metal from accumulating to affect reaction activity. Activated carbon, which is obtained from renewable resources (cellulose, lignin), is a promising material as the catalyst carrier.^16–18^ Activated carbon can provide high specific surface area and wide pore size distribution. Moreover, its inertness, durability and easy recovery of metals are needed for the catalyst. Therefore, some studies on the hydrogenation reaction of furfural are carried out under activated carbon support. In a study by Maryam,^19^ activated carbon as a support was applied for a selective hydrogenation of furfural. They designed a catalyst from activated carbon, which combined all the advantages of both the activated carbon and a monolithic structure in one piece.

Although a large number of studies^20–23^ have achieved a single specific product in the hydrogenation reaction of furfural on their modified catalyst, there are still a few experiments exploring greener catalysts to obtain more efficient conversion rates and higher yields. Therefore, in this work we designed a series of bimetal nickel (Ni) cobalt (Co) catalysts with lignin-based activated carbon as a carrier for furfural reaction to get a higher yield of the target product, named Ni_*x*_Co_*y*_/AC (*x*,*y* represent the Ni and Co loading amount). It is worth mentioning that the support in the catalyst comes from the previous work of the research group and is activated from biomass waste lignin. In order to compare the impact of the carrier on the performance of the catalyst, the carrier also used the biomass full-component raw material corncob and fossil resource coal. We explored the performance of single metals Ni, Co and bimetal in the reaction. Ni_3_Co_1_/AC catalysts, which exhibited a conversion of nearly 100%, were found to be an optimal catalyst for the aqueous selective hydrogenation of furfural to CPL. More significantly, the catalyst still retained most of its quality and activity after five repeated cycles. At last, all of the catalysts were characterized by XRD, XPS, ICP, BET, TEM for theoretical analysis of experimental results.

## 2 Experimental

### 2.1 Materials

The all chemical reagents were purchased and used directly without any treatment: 2-furaldehyde was chose from Shanghai Aladdin Biochemical Technology Co., Ltd; nickel(ii) nitrate hexahydrate (Ni(NO_3_)_2_·6H_2_O) and cobalt(ii) nitrate hexahydrate (Co(NO_3_)_2_·6H_2_O) was purchased from Sinopharm Chemical Reagent Co., Ltd; phosphoric acid was used from Xilong Scientific Co., Ltd; enzymatic hydrolysis of lignin, acid hydrolyzed of lignin and corn cob were provided by Shandong Longlive Bio-technology Co., Ltd; Xiaoquan coal was bought from Ningxia, China. All the solvents (methanol, ethanol and isopropyl alcohol) were obtained from Sigma-Aldrich Company Ltd. Deionized water was applied for all experiments.

### 2.2 General procedure for FFA hydrogenation

In the reaction, 100 mg of furfural, 10 mg of catalyst (10%) and an appropriate amount of deionized water were put into the micro reactor (YZPR-**A 25 mL from Shanghai Yanzheng Experimental Instrument Co., Ltd), and hydrogen was passed through it three times to eliminate the existing air (99.99%) in the reactor. The reaction was then carried out at the desired temperature and stirred at 600 rpm. At the end of the reaction, the reactor was cooled to room temperature and the pressure was released, the resulting mixture was filtered to remove the catalyst, and the filtrate was analyzed by gas chromatography/mass spectrometer using *n*-dodecane as an internal standard. The collected catalyst was washed 3 times with ethanol and dried at 100 °C for the cycle test. The conversion rate of furfural and the product yield were calculated according to the following formula:1
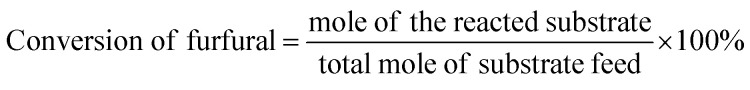
2
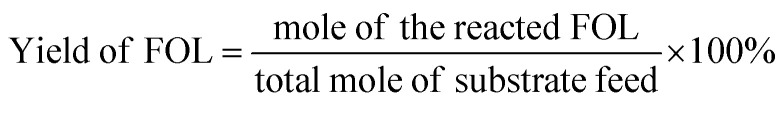
3
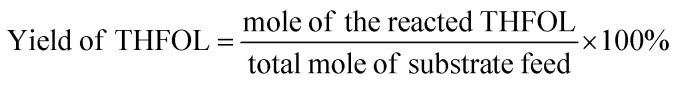
4
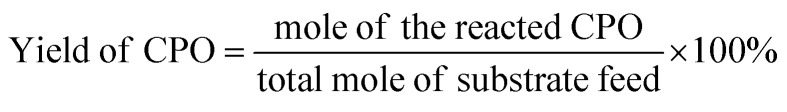
5
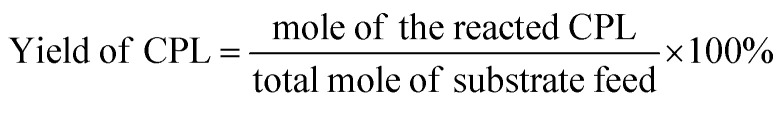


### 2.3 Catalyst preparation

#### 2.3.1 Preparation of the AC

**Scheme 1:**
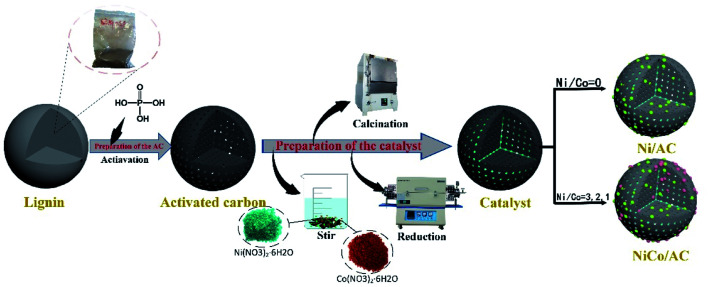
Schematic illustration of the preparation of the Ni_*x*_Co_*y*_/AC catalyst.

The activated carbon was prepared by the phosphoric acid activation method, using enzymatic hydrolysis of lignin, acid hydrolyzed of lignin, corn cob and Xiaoquan coal as raw material, named ELAC, ALAC, CCAC, XCAC, respectively. The specific operation process was as shown in Scheme 1 phosphoric acid and raw material were mixed at the ratio of 3 : 1 (mass ratio), then stirred at room temperature until well mixed. After standing for a while, reaction mixture was put it in a blast drying box at the temperature of 90 °C for 12 h. After taking it out, it was activated at the temperature of 563 °C (heating rate at 5 °C min^−1^) in a muffle furnace and naturally cooled to room temperature. Finally, it was rinsed with boiled deionized water for several times until it is neutral, then placed in an oven at 105 °C for drying to obtain activated carbon. Activated carbon of different raw materials were prepared with the same conditions and the same method.

#### 2.3.2 Preparation of the catalyst

The Ni_*x*_Co_*y*_/AC catalyst was prepared by the impregnation method as shown in Scheme 1. In a typical preparation method of 20% Ni_3_Co_1_/AC (*x*% Ni_3_Co_1_/AC, *x* represents the all metal loading amount) catalyst, Ni(NO_3_)_2_·6H_2_O (1.486 g), Co(NO_3_)_2_·6H_2_O (0.494 g) and AC (2.0 g) were dissolved in 30 mL of deionized water and stirred for 24 hours until uniformly dispersed. The obtained suspension was dried in an oven at 105 °C for 12 hours to remove water. Subsequently, the black solid obtained was calcined in a muffle furnace at 400 °C for 2 h. Finally, it was reduced in a tube furnace at 500 °C for 2 h in a hydrogen atmosphere. Other catalyst preparation methods were the same as Ni_3_Co_1_/AC catalyst.

### 2.4 Catalyst characterization

X-ray diffraction (XRD) is used for phase identification of catalysts. Using 40 kV Cu Kα radiation, the XRD spectrum was recorded on the D8 focusing diffractometer at a rate of 10.0° min^−1^ in the range of 2*θ* = 10.0–80.

X-ray photoelectron spectroscopy (XPS) was performed on Thermo ESCALAB 250XI. An aluminum anode (Al Kα = 1486.6 eV) was used to collect spectra at 14.8 kV and 4.5 mA. The pass energy of the analyzer was 100 eV for the wide scan and 30 eV for the narrow scan. The energy correction is based on the C 1s peak of indeterminate carbon at 284.5 eV. Use Advantage software to install XPS mode.

The pore structure data used Micrometric ASAP 2020 to analyze the catalyst N_2_ adsorption isotherm at −196 °C. Before the measurement, the catalyst was degassed in vacuum at 200 °C for 10 hours. The BET surface area is calculated based on the linear part of the BET diagram method. The amount of Ni, Co in the catalysts was identified by inductively coupled palsma-optical emission spectroscopy (ICP-OES) on a PerkinElmer 8300 instrument.

Transmission electron microscope (TEM) images were obtained using FEI Tecnai F20 operating at 200 kV. First, the catalyst sample was ground, using ethanol as the solvent. After sonication, the suspension was dropped onto the copper grid coated with carbon film. The particle size distribution is calculated by measuring the size of more than 200 random particles in the TEM image by ImageJ software.

## 3 Results and discussion

### 3.1 Characterization of the catalysts

**Fig. 1:**
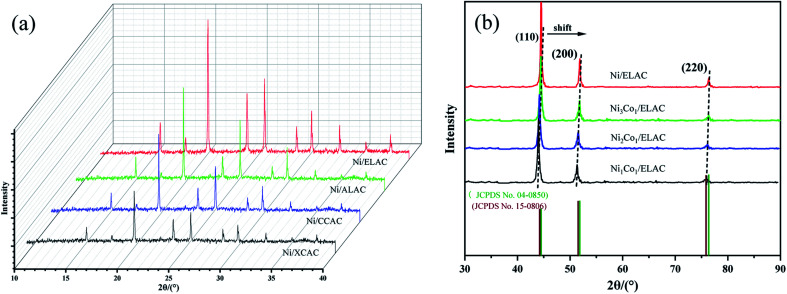
XRD patterns of (a) Ni/ELAC, Ni/ALAC, Ni/CCAC and Ni/XCAC catalysts (b) Ni/ELAC, Ni_3_Co_1_/ELAC, Ni_2_Co_1_/ELAC, Ni_1_Co_1_/ELAC catalysts.

Firstly, the powder X-ray diffraction (XRD) determined the phase characteristics of the catalyst including different supports (ELAC, ALAC, CCAC, XCAC) and ratio of metal nickel and cobalt (Ni_3_Co_1_, Ni_2_Co_1_, Ni_1_Co_1_) in the system for the hydrogenation of furfural. Fig. 1a exhibited the XRD patterns of different carriers (Ni/ELAC, Ni/ALAC, Ni/CCAC, Ni/XCAC). Peak shapes of them were similar overall, because the preparation of activated carbons was all activated by phosphoric acid, the various peaks could be different states of the phosphoric acid–biopolymer complex. Different metal ratio catalysts (Ni_3_Co_1_/ELAC, Ni_2_Co_1_/ELAC, Ni_1_Co_1_/ELAC) were shown in Fig. 1b. The diffraction pattern for the catalysts had three broad peaks at around 44.50, 51.85 and 76.37, corresponding to (111), (200) and (220) of the nickel (JCPDS no. 04-0850), respectively. In addition, the pattern of the catalyst with the increasing of the cobalt loading could be found to move a little to the left, this could be ascribed to the comparable structural features of nickel and cobalt, which had a number of peaks at around 44.22, 51.52 and 75.85 according to JCPDS card no. 15-0806. Through the analysis of XRD, it was proved that the metal nickel and cobalt were successfully supported on the catalyst.

**Fig. 2:**
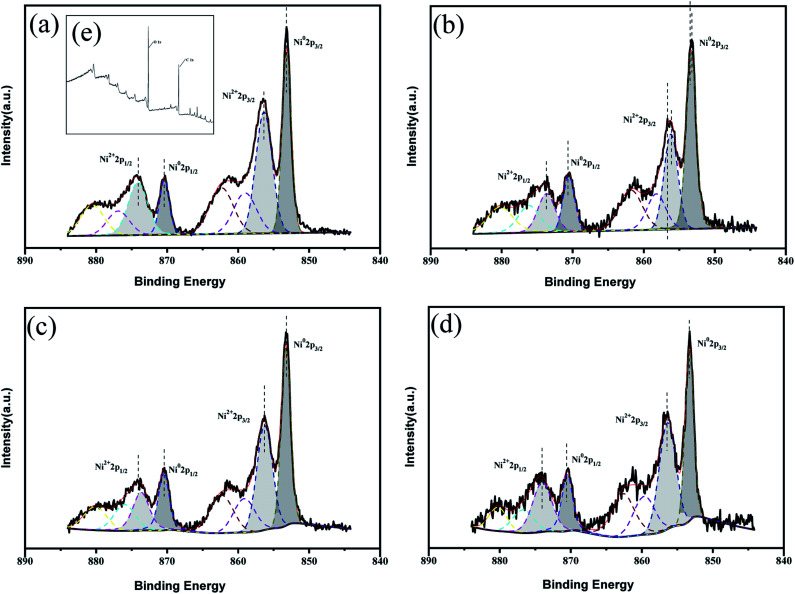
XPS patterns of Ni 2p over (a) Ni/ELAC, (b) Ni_3_Co_1_/ELAC, (c) Ni_2_Co_1_/ELAC, (d) Ni_1_Co_1_/ELAC catalysts, (e) XPS patterns of Ni/ELAC catalyst.

**Fig. 3:**
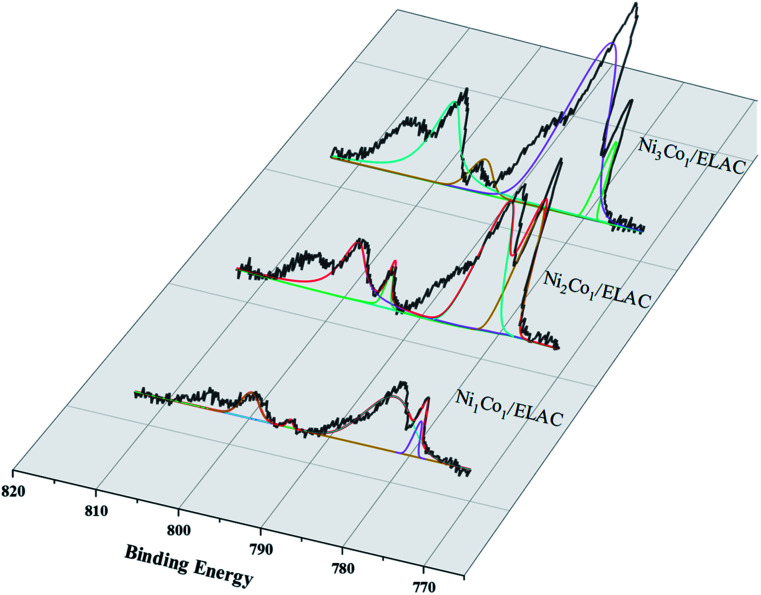
XPS patterns of Co 2p over Ni_3_Co_1_/ELAC, Ni_2_Co_1_/ELAC, Ni_1_Co_1_/ELAC catalysts.

X-ray photoelectron spectroscopy (XPS) was used to carry out surface analysis of the catalysts (Ni/ELAC, Ni_3_Co_1_/ELAC, Ni_2_Co_1_/ELAC, Ni_1_Co_1_/ELAC) and characterize the nickel and cobalt oxidation state. The Ni^0^ 2p spectrum exhibited two contributions, 2p_1/2_ and 2p_3/2_ (resulting from the spin orbit splitting), located at respectively 870 eV and 852.4 eV (Fig. 2a–d), which could be assigned to the presence of metallic nickel. The presence of the O 1s peak (Fig. 2e) and Ni^2+^ (Fig. 2e), which was evidenced by a shoulder observed on the main peak at 873.8 eV (Ni^2+^ 2p_1/2_) and 852.8 eV (Ni^2+^ 2p_3/2_), indicated that oxidation of the samples took place after exposure to the atmosphere. Similarly, the XPS spectrums of Co 2p in catalysts (Fig. 3) exhibited four main peaks with binding energy at 778.45 eV, 782.7 eV, 793.66 eV and 798 eV corresponding to Co^0^ 2p_3/2_, Co^2+^ 2p_3/2_, Co^0^ 2p_1/2_ and Co^2+^ 2p_1/2_. In addition, an increase in the binding energy shift with increasing loading of cobalt had been observed for Ni_1_Co_1_/ELAC in accordance with Ni/ELAC with a maximum shift of around 0.3 eV. When comparing these samples, the shift to higher binding energy from Ni/ELAC to Ni_1_Co_1_/ELAC provided evidence of an interaction between the nickel, cobalt and support species.

The physicochemical properties of AC supported Ni/Co-based catalysts were listed in Table 1. Firstly, ICP analysis showed though the metal nickel and cobalt content was somewhat lost (theoretical content value 20%), the measured bimetal ratio was very close to the theoretical design value, indicating metal nickel and cobalt could be effectively attached to the surface of activated carbon and the porous material activated carbon was feasible and effective as a metal support. In addition, four different activated carbon supported catalysts (Table 1 row 2–5) had different specific surface areas and pore structures, which were determined by the carrier. Bimetallic nickel and cobalt catalyst exhibited better BET surface and pore volume comparing to the single metal nickel catalyst. With the increase of another metal cobalt, the bimetallic catalyst had larger specific surface area and smaller average pore size. Similar studies from Xu's group had also confirmed that the increase in cobalt content would reduce the average pore size.^24,25^ The results (Table 1 row 2, 6–8) proved that the catalyst with the addition of another metal cobalt would lead to the improved dispersibility, which finally caused the increase in specific surface area and decrease in average pore size.

**Table 1:** Chemical and physical properties of Ni_*x*_Co_*y*_/AC catalysts

Catalysts	Composition^a^ (wt%)			*S*_BET_ b (m^2^ g^−1^)	*V*_p_ b (cm^3^ g^−1^)	Average pore diameter^b^ (nm)
	Ni	Co	Ni/Co			
Ni/ELAC	18.79	—	—	354.151	0.311	3.574
Ni/ALAC	18.35	—	—	312.141	0.298	3.784
Ni/CCAC	18.24	—	—	260.335	0.224	3.868
Ni/XCAC	18.16	—	—	236.546	0.214	4.213
Ni_3_Co_1_/ELAC	14.39	4.81	2.99	369.909	0.321	3.482
Ni_2_Co_1_/ELAC	12.26	5.81	2.11	371.253	0.356	3.318
Ni_1_Co_1_/ELAC	9.16	8.64	1.06	375.482	0.374	3.292

a Measured by ICP-AES analysis.

b Evaluated from N_2_ adsorption–desorption isotherms.

**Fig. 4:**
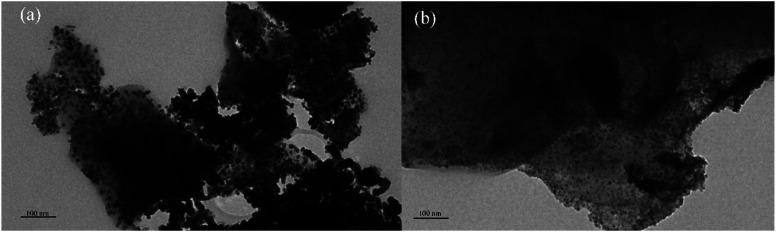
TEM images of (a) Ni/ELAC and (b) Ni_3_Co_1_/ELAC.

The transmission electron microscopy (TEM) was used to analyze the metal dispersion on the catalyst surface more intuitively. Fig. 4 showed TEM micrographs of reduced Ni/ELAC and Ni_3_Co_1_/ELAC catalysts, respectively. It could be seen that metal particles displayed outside the support activated carbon. The main particle sizes over Ni/ELAC and Ni_3_Co_1_/ELAC catalysts were around 12.17 nm and 8.75 nm, respectively, by the calculation of software ImageJ. In comparing with the TEM images of the Ni/ELAC and Ni_3_Co_1_/ELAC catalysts, as could be seen in Fig. 4, the mean metal size decreased while cobalt was added in the Ni_3_Co_1_/ELAC catalyst with the same metal loading amount (20 wt%). The particle sizes were consistent well with the results analyzed by BET in Table 1. The result proved once again that the increase in cobalt content would reduce the average pore size.^26^ In conclusion, bimetallic Ni_*x*_Co_*y*_ catalysts were more suitable for furfural hydrogenation reaction than single metal Ni catalyst.

### 3.2 Optimization of FFA hydrogenation

#### 3.2.1 Effect of different catalysts

The results for hydrogenation of furfural in aqueous solution over different designed Ni_*x*_Co_*y*_/AC catalysts were listed in Table 2. As could be seen in Table 2, the main products after reaction over any catalyst were FOL, CPO, CPL and THFOL. Others could be an intermediate and not presented. The experiment (Table 2 entry 1) was carried out without any catalyst and no product was obtained, demonstrating the reaction must be carried out under the action of a catalyst. When the carrier ELAC was added, the experiment (Table 2 entry 2) still hardly reacted, explaining that the carrier doesn't have much catalytic activity without metal addition. When metallic nickel was added to the carrier activated carbon, the hydrogenation reaction over Ni/ELAC catalyst attained an 78.1% conversion and 80.6% selectivity of CPL, which were higher than those (Table 2 entry 7) obtained with Ni/CNT catalyst^14^ (68.3% and 42.8%, respectively), demonstrating that activated carbon was suitable as the carrier in the reaction of furfural hydrogenation. In order to study the effect of support on the catalytic reaction of furfural hydrogenation, four activated carbons (ELAC, ALAC, CCAC, XCAC) with different raw materials and same preparation methods were selected. The hydrogenation reaction over Ni/ELAC, Ni/ALAC, Ni/CCAC, Ni/XCAC catalysts (Table 2 entry 3–6) attained conversions of 78.1%, 69.4%, 44.2%, 34.1% and CPL selectivity of 80.6, 74.7, 45.5, 40.1%, respectively. This result may be related to different surface area and pore structure of activated carbon. A larger specific surface area was more beneficial to metallic nickel adhesion on the carrier and the progress of the reaction.^27,28^ It could also be proved from the BET data (Table 1) that Ni/ELAC had the largest specific surface area (354.151 m^2^ g^−1^) over four catalysts.

**Table 2:** Hydrogenation of furfural over various catalysts^a^

Entry	Catalyst	FFA con. (%)	Selectivity (%)				
			FOL	CPO	CPL	THFOL	Others
1^b^	None	0	0	0	0	0	0
2^b^	ELAC	8.3 ± 1.13	<10.0	0	0	0	<3.0
3^b^	Ni/ELAC	78.1 ± 1.47	2.9	7.6	80.6	1.1	2.2
4^b^	Ni/ALAC	69.4 ± 1.52	4.3	10.8	74.7	2.5	4.1
5^b^	Ni/CCAC	44.2 ± 1.66	1.3	12.5	45.5	1.9	3.6
6^b^	Ni/XCAC	34.1 ± 1.98	2.8	13.5	40.1	2.2	3.5
7^c^	Ni/CNT	68.3 ± 2.02	0.6	17.3	42.8	0.4	7.2
8^b^	Ni_3_Co_1_/ELAC	100.0 ± 0.71	2.0	2.0	94.1	0.8	1.2
9^b^	Ni_3_Co_1_/ALAC	83.4 ± 2.48	3.2	5.3	70.4	2.3	3.4
10^b^	Ni_2_Co_1_/ELAC	94.5 ± 1.14	2.4	4.8	89.2	0.7	2.2
11^b^	Ni_2_Co_1_/ALAC	76.8 ± 1.58	3.7	6.4	67.2	1.0	5.4
12^b^	Ni_1_Co_1_/ELAC	90.7 ± 1.69	3.1	7.1	85.4	0.4	2.5
13^b^	Ni_1_Co_1_/ALAC	74.8 ± 2.54	4.1	9.0	65.4	0.8	6.6

a Reaction conditions: furfural, 100 mg; catalyst, 10 mg; H_2_O, 10 mL; temperature, 140 °C.

b Time, 2 h; initial H_2_ pressure, 1.0 MPa.

c Time, 6 h; initial H_2_ pressure, 4.0 MPa.

Subsequently, two optimal activated carbons (ELAC, ALAC) were selected in the experiment, and the influence of bimetallic addition on the experimental results was explored. By analyzing experimental results (Table 2 entry 3, 8, 10, 12) over Ni/ELAC, Ni_3_Co_1_/ELAC, Ni_2_Co_1_/ELAC, Ni_1_Co_1_/ELAC catalysts, we could find that bimetal supported catalysts could achieve higher conversion and selectivity, ranking Ni_3_Co_1_/ELAC > Ni_2_Co_1_/ELAC > Ni_1_Co_1_/ELAC > Ni/ELAC. We chose another carrier (ALAC) for experiment (Table 2 entry 4, 9, 11, 13) and got the same conclusion. This might be due to the fact that bimetallic ions could more fully utilize the pore structure of the support for catalytic reactions.^26^

By choosing the catalyst carrier and metal loading ratio optimization, Ni_3_Co_1_/ELAC catalyst was the most suitable catalyst among all selected catalysts in the furfural hydrogenation reaction for conversion of nearly 100% and CPL selectivity of 94.1%. In summary, Ni_3_Co_1_/ELAC was selected for follow-up research.

#### 3.2.2 Effect of reaction time, H_2_ pressure and temperature over Ni_3_Co_1_/ELAC catalyst

**Fig. 5:**
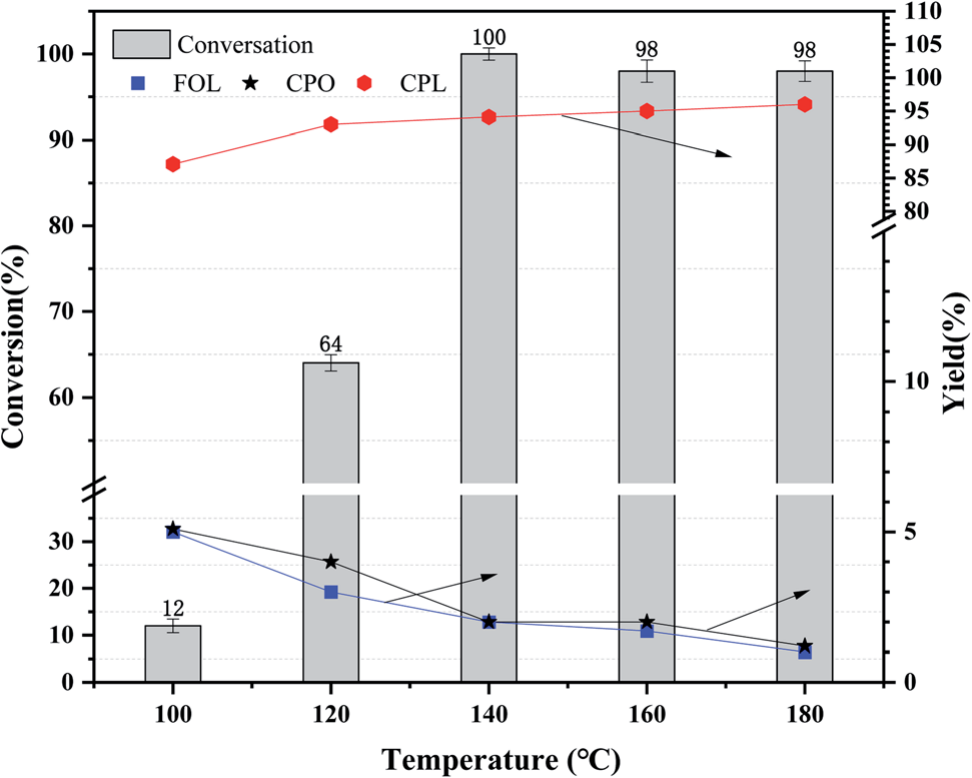
Effects of reaction temperature over Ni_3_Co_1_/ELAC catalyst in furfural hydrogenation (initial H_2_ pressure: 1 MPa; reaction time: 2 h).

**Fig. 6:**
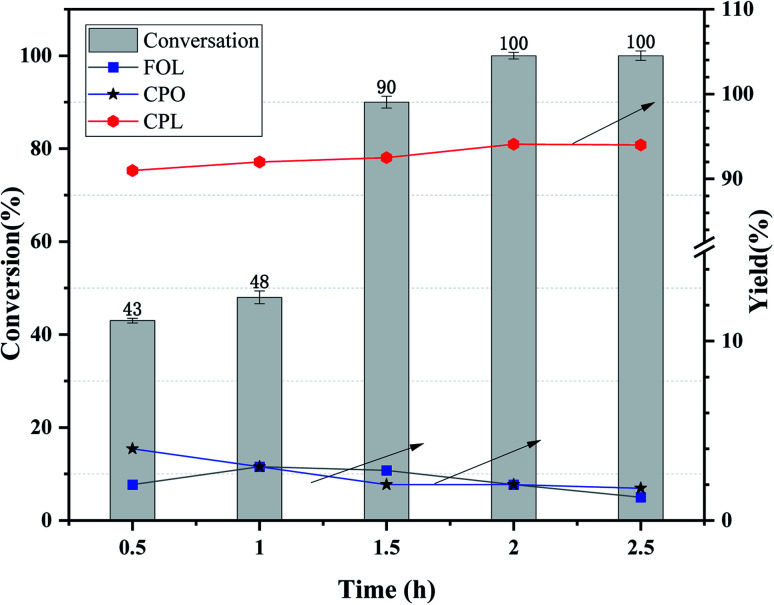
Effects of reaction time over Ni_3_Co_1_/ELAC catalyst in furfural hydrogenation (reaction temperature: 140 °C, initial H_2_ pressure: 1 MPa).

**Fig. 7:**
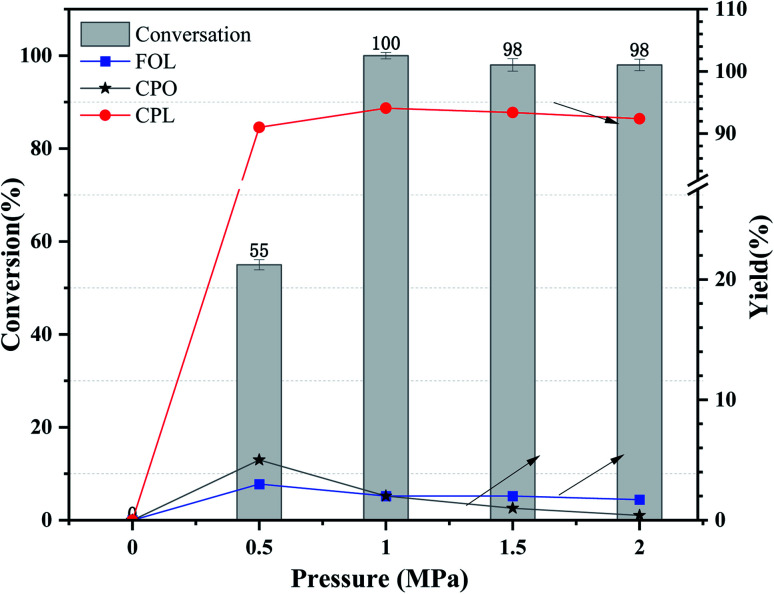
Effects of initial H_2_ pressure over Ni_3_Co_1_/ELAC catalyst in furfural hydrogenation (reaction temperature: 140 °C, reaction time: 2 h).

In this part, the parameters of furfural hydrogenation in the aqueous over Ni_3_Co_1_/ELAC, including the reaction temperature, initial H_2_ pressure, and the reaction time, were explored and the results were presented in Fig. 5–7. To analyze the catalyst reaction effect over different reaction temperature, the furfural hydrogenations were conducted at 100 °C, 120 °C, 140 °C, 160 °C, 180 °C, respectively, with an initial H_2_ pressure of 1 MPa and the reaction time of 2 h. As was shown in Fig. 5, it could be firstly found that reaction temperature had a great effect on the conversion of furfural, which improved from 12% to about 100% while the temperature increased, demonstrating that higher temperature promoted the cleavage reaction in the furfural molecule and the following formation of new C–C bond.^29^ The results were proved in previous studies.^30^ The conversion rate of furfural reached the peak (about 100%) at 140 °C, and then decreased slowly with the increase of temperature. The conversion of furfural exhibited a similar trend when the temperature increased comparing with the Zhou's studies,^31^ which is carried out over Cu_*x*_Zn_*y*_/CNT catalyst. On the other hand, it was surprising to find that, when product distribution was noticed, at different temperatures, the main products turned out to be CPL (at least 90% yield), showing Ni_3_Co_1_/ELAC catalyst excellent selectivity in the hydrogenation of furfural. And the temperature increase improved the yield of CPL and decreased the yield of CPO, FOL. Considering the higher conversion and lower reaction temperature, 140 °C was regarded as the optimal result.

As is known to us, the reaction temperature, the initial H_2_ pressure and the reaction time are extremely important factors for the hydrogenation of furfural.^32,33^ Herein, the effect of reaction time over Ni_3_Co_1_/ELAC catalyst in reaction temperature of 140 °C and initial H_2_ pressure of 1 MPa was also investigated in Fig. 6. We conducted the reaction time of furfural hydrogenation from 0.5 h to 2.5 h, the results turned out to be an upward trend from 43% to 100% in the conversion of furfural. However, when the reaction time was less than 1 h or the reaction time was more than 1.5 h, the furfural conversion increased slowly and the change was not obvious. Larger furfural conversion occurred when the reaction time increased from 1 h to 1.5 h because the conversion of furfural to CPL contained several steps, enough time was necessary to perform this selective reaction furfural to CPL. When the reaction time extended to 2 h, nearly 100% conversion was obtained. So 2 h was the final optimal reaction time after the inspection.

The influence of initial H_2_ pressure was also studied at a range of 0 MPa to 2 MPa over Ni_3_Co_1_/ELAC in reaction temperature of 140 °C and the reaction time of 2 h, which was obviously lower compared with previous studies.^31^ It could be clearly observed in Fig. 7 that no reaction occurred while no hydrogen was added in the system. The conversion of furfural and the yield of CPL significantly increased from 55% to nearly 100% and from 91% to 94%, respectively, with the increase of the initial H_2_ pressure from 0.5 MPa to 1 MPa, revealing that appropriate hydrogen was beneficial to the selective conversion of furfural to CPL in the aqueous. As the initial H_2_ pressure improved from 1 MPa to 2 MPa, there was almost no change in conversion and the yield of CPL. In compared with previous studies,^34–36^ we also discovered that extra hydrogen might lead to the decrease of main product (CPL) and the increase of derivates (THFOL). Through the consideration and analysis of three important variable factors (reaction temperature, reaction time, initial H_2_ pressure), we had obtained the optimal conditions (reaction temperature: 140 °C, reaction time: 2 h, initial H_2_ pressure: 1 MPa) in furfural hydrogenation over Ni_3_Co_1_/ELAC catalyst.

### 3.3 Scope of the substrates and mechanism of furfural hydrogenation

Although many previous studies have proved the path of the hydrogenation reaction of furfural, the different choice of catalyst will produce different pathway and desired chemicals. In order to infer a plausible reaction mechanism in this paper, we compared the results (Table 3) of using different substates, which had a similar basic furan structure, under the same catalyst and hydrogenation reaction atmosphere. The experiments were carried out at the reaction time of 2 h, the reaction temperature of 140 °C and the initial H_2_ pressure of 1 MPa using Ni_3_Co_1_/ELAC catalyst as optimum catalyst. Compared with furfural, 2-acetylfuran, which was only added a methyl group in the carbonyl carbon position, obtained the product (2-methyl cyclopentanone), also correspondingly a methyl group at the same position as shown in Table 3 entry 2. The difference was that the yield had declined to 92%. But when choosing 5-methylfurfural or 5-(hydroxymethyl)furfural, the rearrangement reaction of the furan ring could not take place (Table 3 entry 3–4). The products we got were (5-methylfuran-3-yl)methanol and 2,5-bis(hydroxymethyl)furan, and the corresponding yields were 86% and 84%.

**Table 3:** Effects of derivatives over Ni_3_Co_1_/ELAC catalyst in furfural hydrogenation^a^

Entry	Substrate	Main product	Yield (%)
1	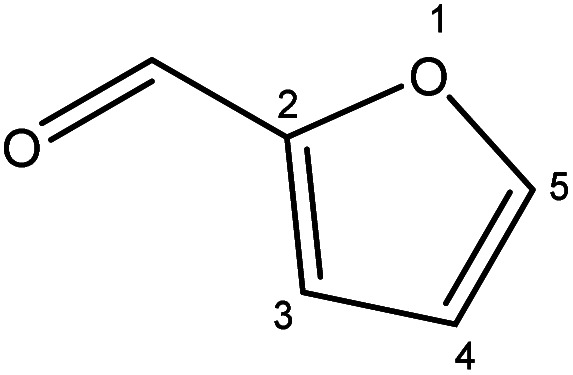	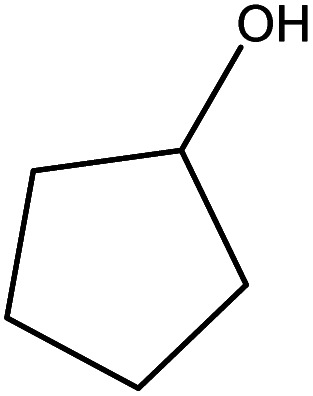	94.1
2	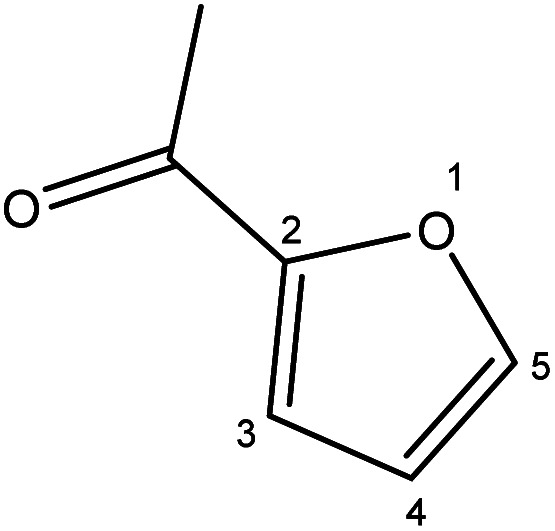	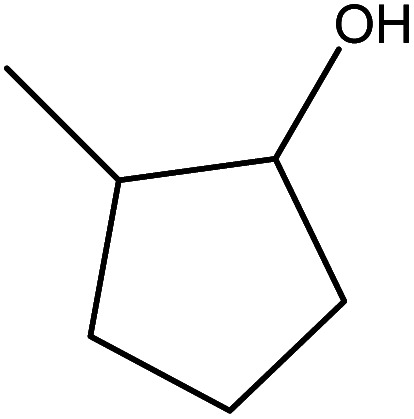	92.3
3	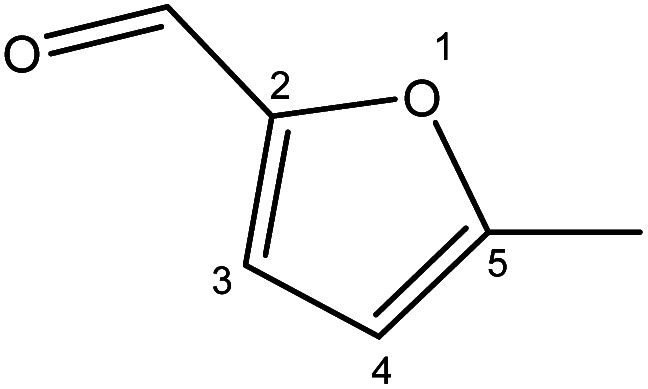	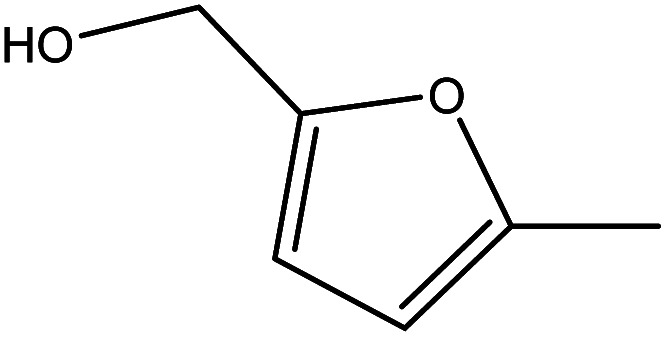	86.2
4	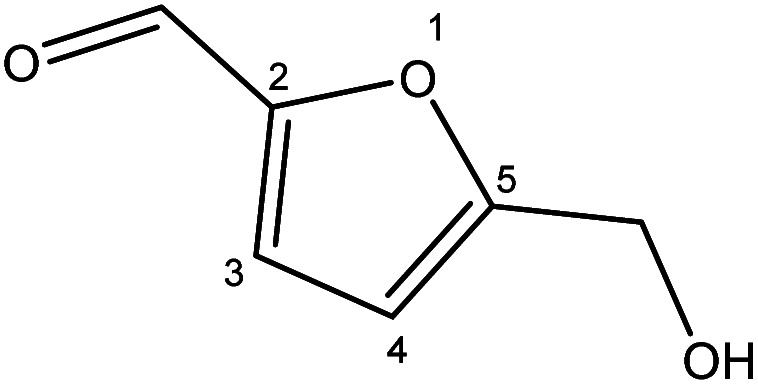	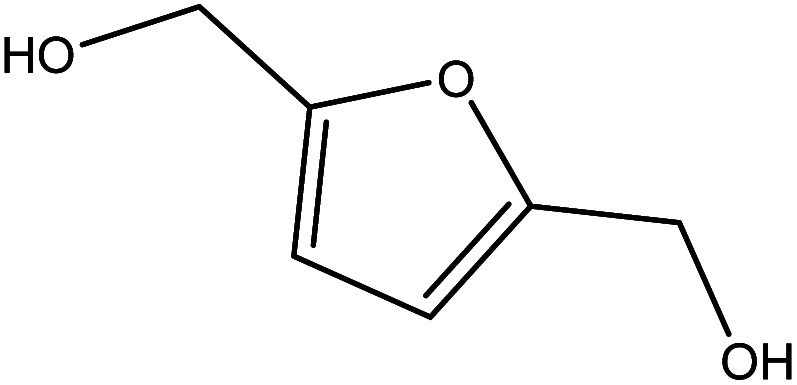	85.7

a Reaction conditions: reaction temperature, 140 °C; reaction time, 2 h; initial H_2_ pressure, 1 MPa.

**Scheme 2:**
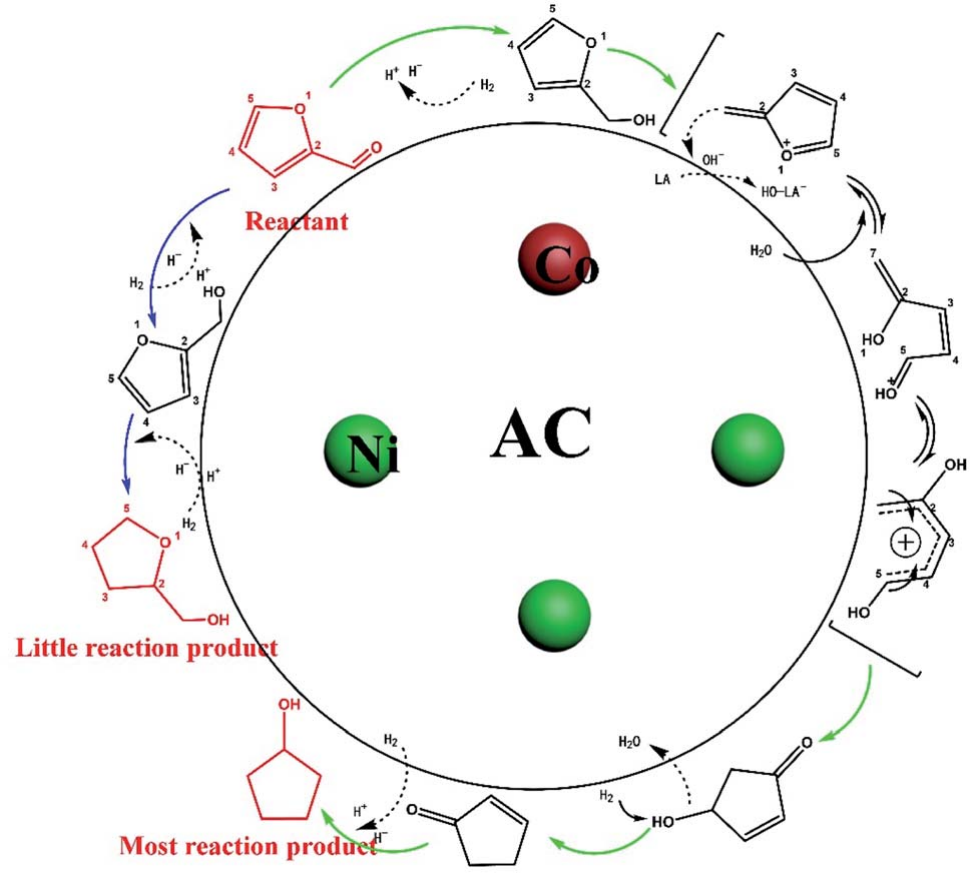
The reaction process of the furfural hydrogenation over Ni_3_Co_1_/ELAC catalyst.

**Fig. 8:**
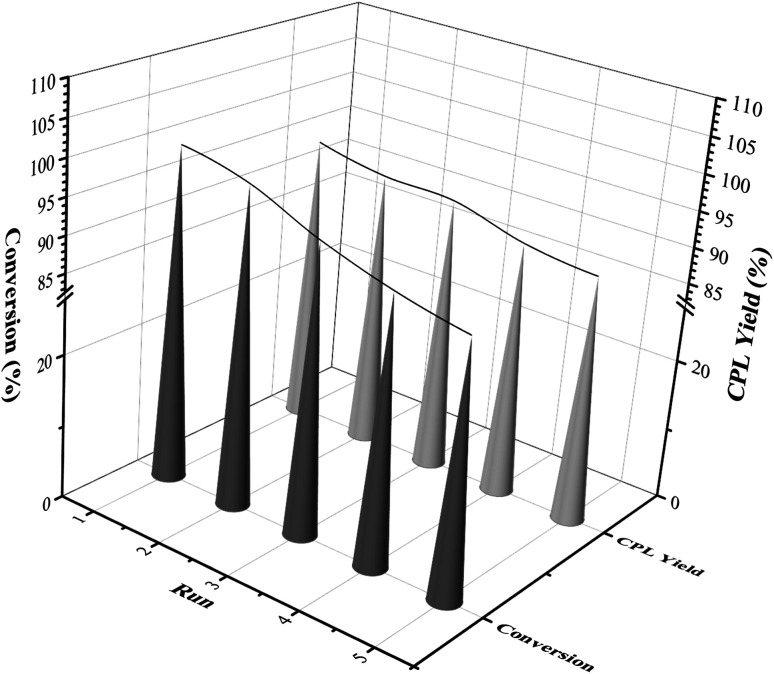
Reuse of the Ni_3_Co_1_/ELAC catalyst in furfural hydrogenation.

In this work, CPL occupied the largest proportion as the main product. And some byproducts including FOL,^37^ THFOL,^38^ CPO,^39^*etc.*, which might be used as an important product for other people's research, might only be intermediate products in the reaction path of the target product of CPL, or even be generated under other irrelevant reaction paths. The intermediates were not easy to capture due to the rapid reaction, so we performed an experiment at low rate, which was carried out at 80 °C for 30 min.^40^ In addition to the substances that appeared in the previous reaction with a low conversion, we also detected 2-cyclopentenozane, showing that they are in an unstable state in reaction. Based on previous reports and some detected intermediates, the proposed mechanism of the hydrogenation of furfural over Ni_3_Co_1_/ELAC was presented in Scheme 2. FOL might be the first to be formed by the reaction as hydrogen atoms attacked the carbonyl group on the surface of furfural, which happened quickly and continued to happened other different reactions, resulting in the little yield of FOL. This step was affected by many factors such as temperature, H_2_ pressure, time, and catalysts by Fig. 6–8. THFOL could be generated by all the double bonds on the FOL ring with hydrogen^11^ (Scheme 2 blue arrow). However, another reaction path (Scheme 2 red arrow), water molecules participated in, would eventually generate CPL by the rearrangement of the ring. FOL removed hydroxide ions under the action of Lewis acid, and molecular rearrangement reaction occurred under the attack of water molecules to form 4-hydroxy-2-cyclopentenone. Then CPL was generated under the action of hydrogen. It was found through the study of Yang that this reaction occurred in a large amount in organic solution, and the low yield in the water phase.^10,41^ This paper in the water phase also proved this conclusion. In order to verify this conclusion, we tried to choose different solvents (methanol, ethanol, isopropanol, water) for the reaction. During the data collection (Table 4), when methanol, ethanol, isopropanol acted as a solvent in the reaction, almost no CPL was obtained and the dominate product was THFOL. The results proved that the production of CPL required the rearrangement reaction and the participation of water molecules.

**Table 4:** Effects of solvents over Ni_3_Co_1_/ELAC catalyst in furfural hydrogenation^a^

Solvent	Conversion (%)	THFOL yield (%)	CPL yield (%)
Methanol	98.7	87.1	1.1
Ethanol	98.2	86.4	0.4
Isopropanol	99.8	94.2	0.5
Water	100.0	0.8	94.1

a Reaction conditions: reaction temperature, 140 °C; reaction time, 2 h; initial H_2_ pressure, 1 MPa.

From the perspective of economy and environmental protection, it was necessary to test the cycle activity of the Ni_3_Co_1_/ELAC catalyst in the furfural hydrogenation. After each reaction, the catalyst was separated from the reaction mixture by centrifugation, followed by washing with ethanol for several times and drying for the next reaction. The results were shown in Fig. 8. In the first three cycles of the reaction, the conversion rate of furfural and the selectivity of CPL remained almost the same as the previous two reactions. After recycling five times, the conversion rate of furfural and the selectivity of CPL decreased by 11% (from 100% to 89%) and 5.8% (from 94.1% to 88.3%), respectively. The Ni_3_Co_1_/ELAC catalyst in this paper showed good stability during the reactions, which was likely attributable to strong interactions between the support activated carbon and the metal composition nickel and cobalt.

## 4 Conclusion

In general, we have designed a simple strategy to effectively prepare the Ni_3_Co_1_/ELAC catalyst, which can effectively convert furfural (100% conversion) into cyclopentanol (94.1% selectivity). Compared with other studies (Table 5), the catalyst showed high selectivity for cyclopentanol. Experiments have found that the catalyst support has an impact on the conversion of furfural, which may be related to the pore structure of the support. So, activated carbon with suitable pore size for the loading of Ni–Co catalyst was prepared by use of enzymatic lignin as raw material and phosphoric acid activation. Bimetal ratio and reaction conditions (reaction temperature, reaction time, initial H_2_ pressure) are important factors that affect experimental results in the furfural hydrogenation. For the formation of the product CPL in the hydrogenation of furfural, the rearrangement reaction under the action of water molecules and Lewis acid are required.

**Table 5:** Performance comparison with reported catalysts

Entry	Catalyst	FFA con. (%)	Selectivity (%)				
			FOL	CPO	CPL	THFOL	Others
1	Ni_3_Co_1_/ELAC	100	2.0	2.0	94.1	0.8	1.2
2 (ref. 42)	Ni_1_Co_3_/γ-Al_2_O_3_	100	13.2	42.1	11.5	0.4	—
3 (ref. 43)	Ni_3_Co_1_/CNTs	94	2	7	88	2	1
4 (ref. 44)	Cu_0.4_Mg_5.6_Al_2_	100	—	—	98.6	—	—
5 (ref. 45)	Pd/MgAlO_*x*_	62.9	48.6	—	—	32.4	—
6 (ref. 46)	Pt/SiO_2_ + ND_2_O_3_	—	—	88	0	—	—

## Conflicts of interest

There are no conflicts to declare.

## Supplementary Material
